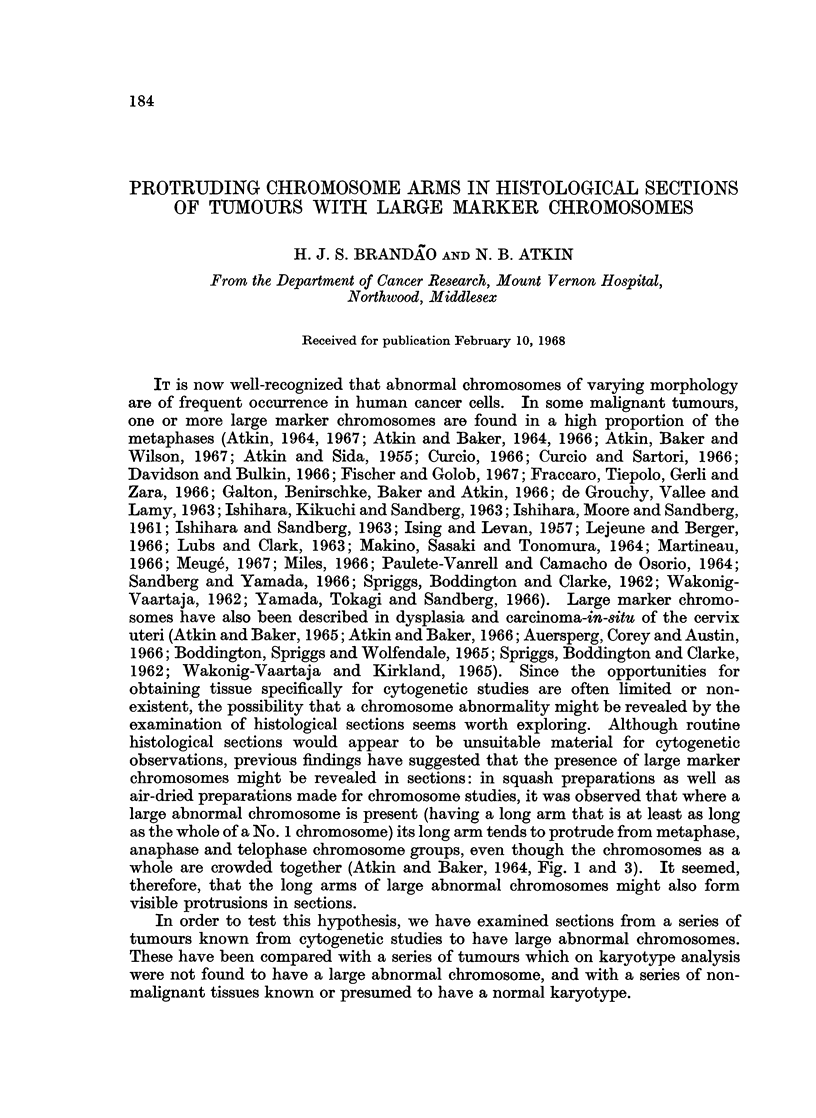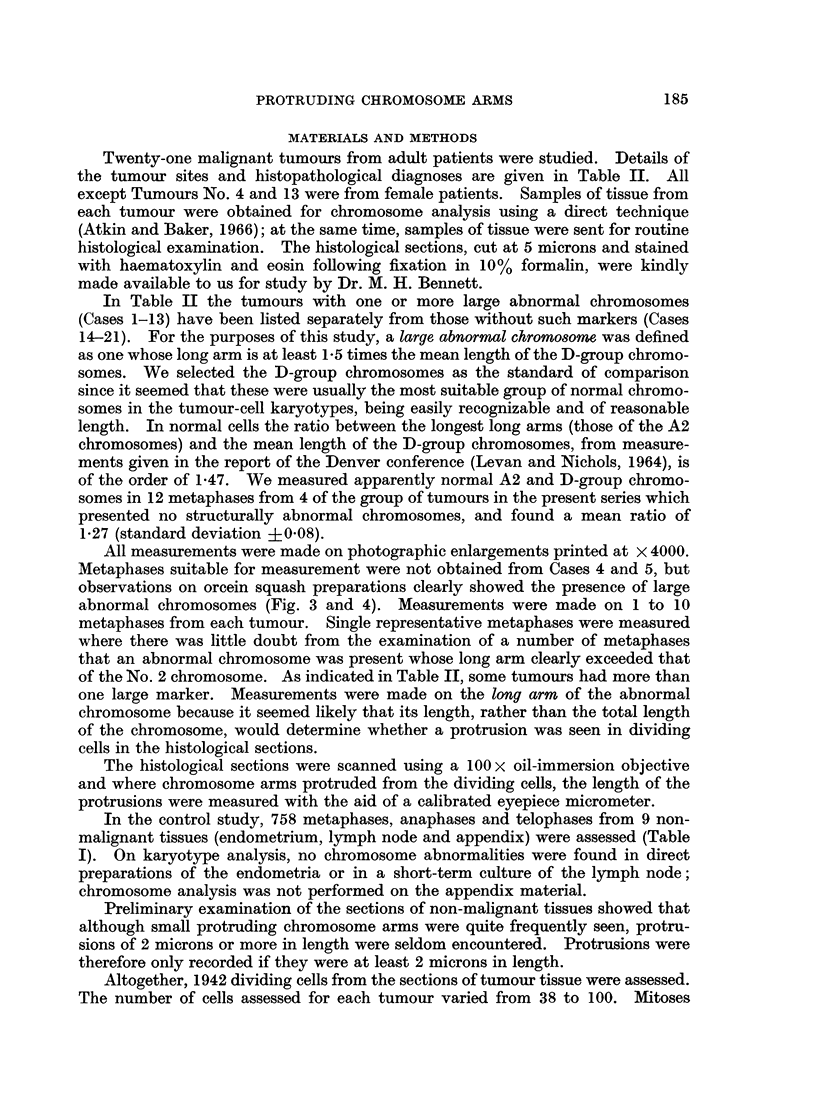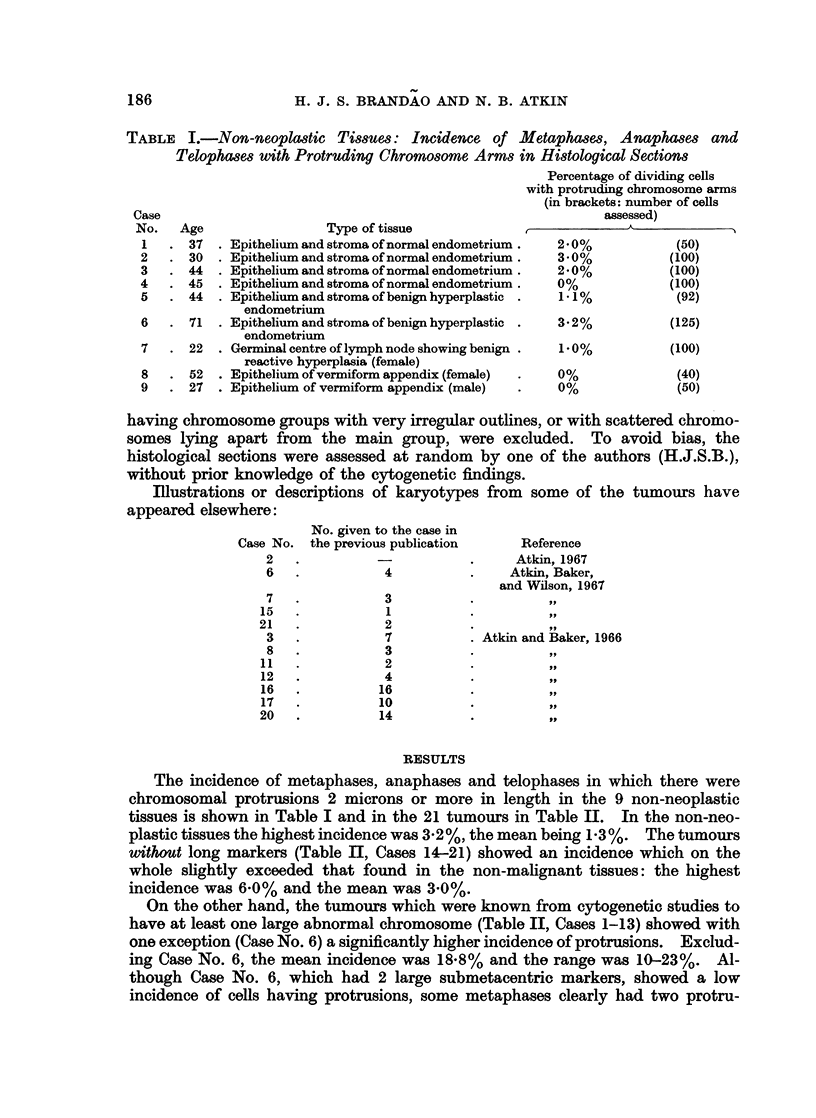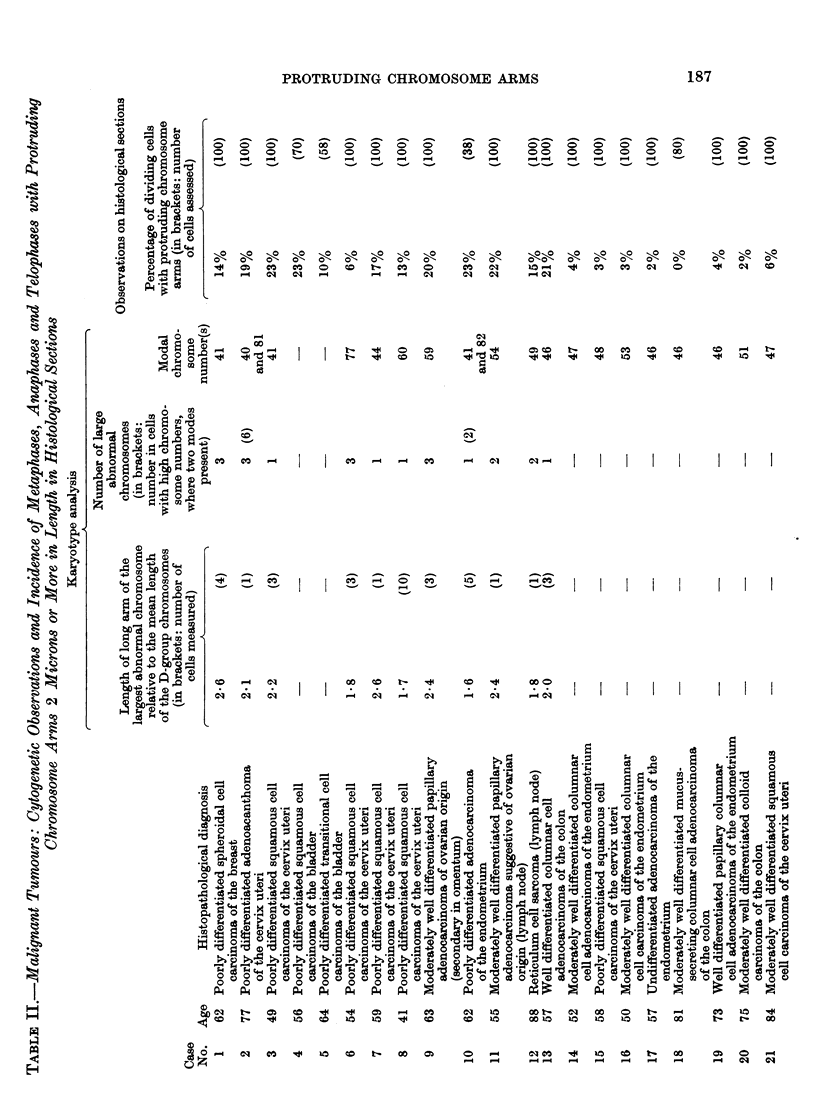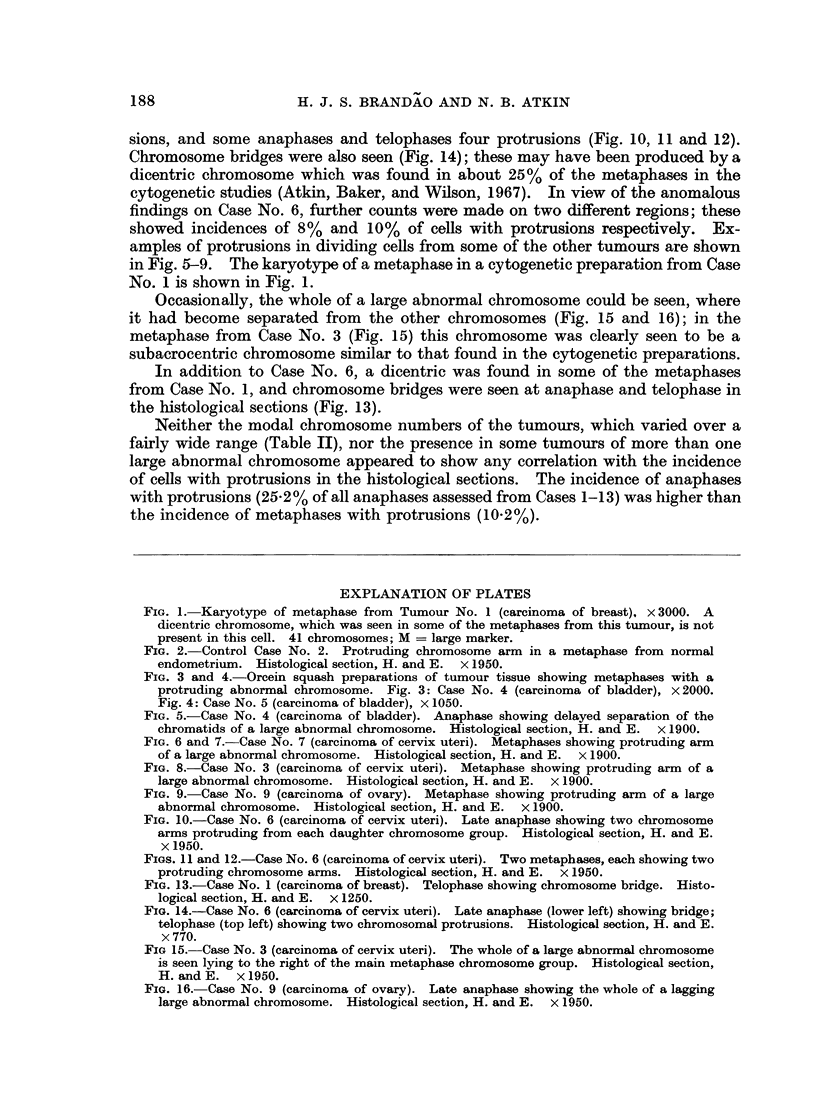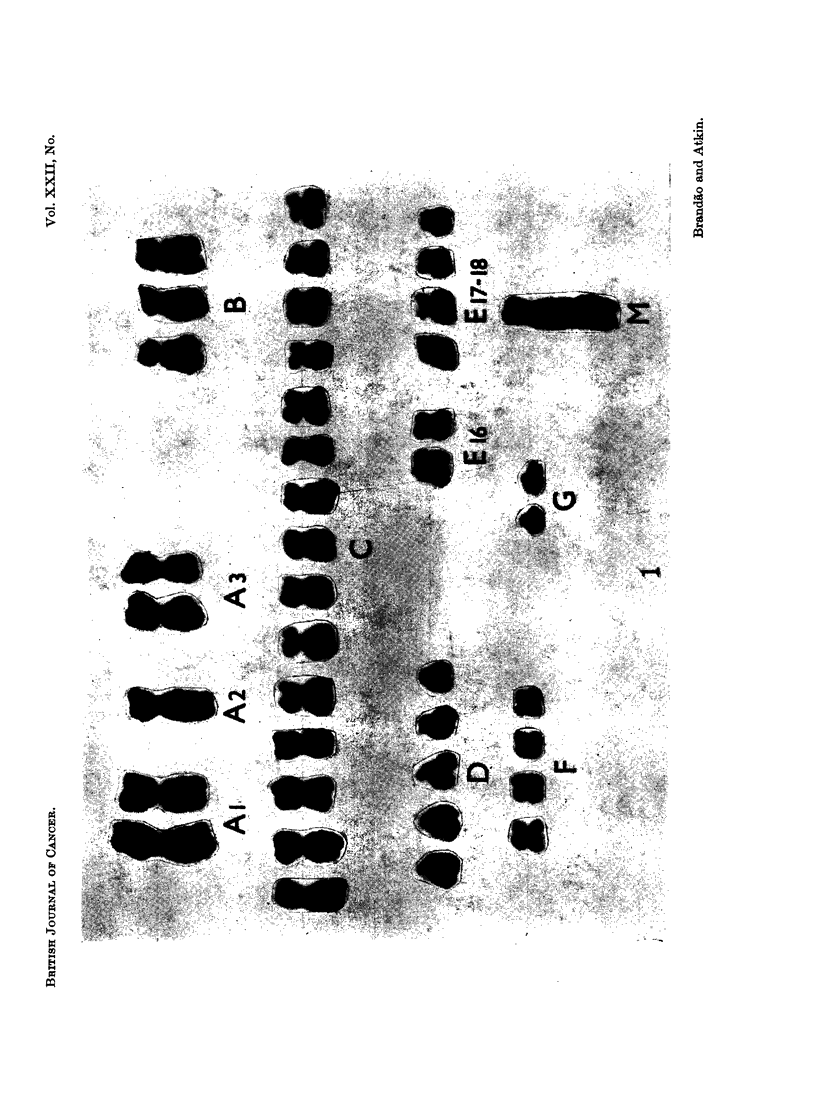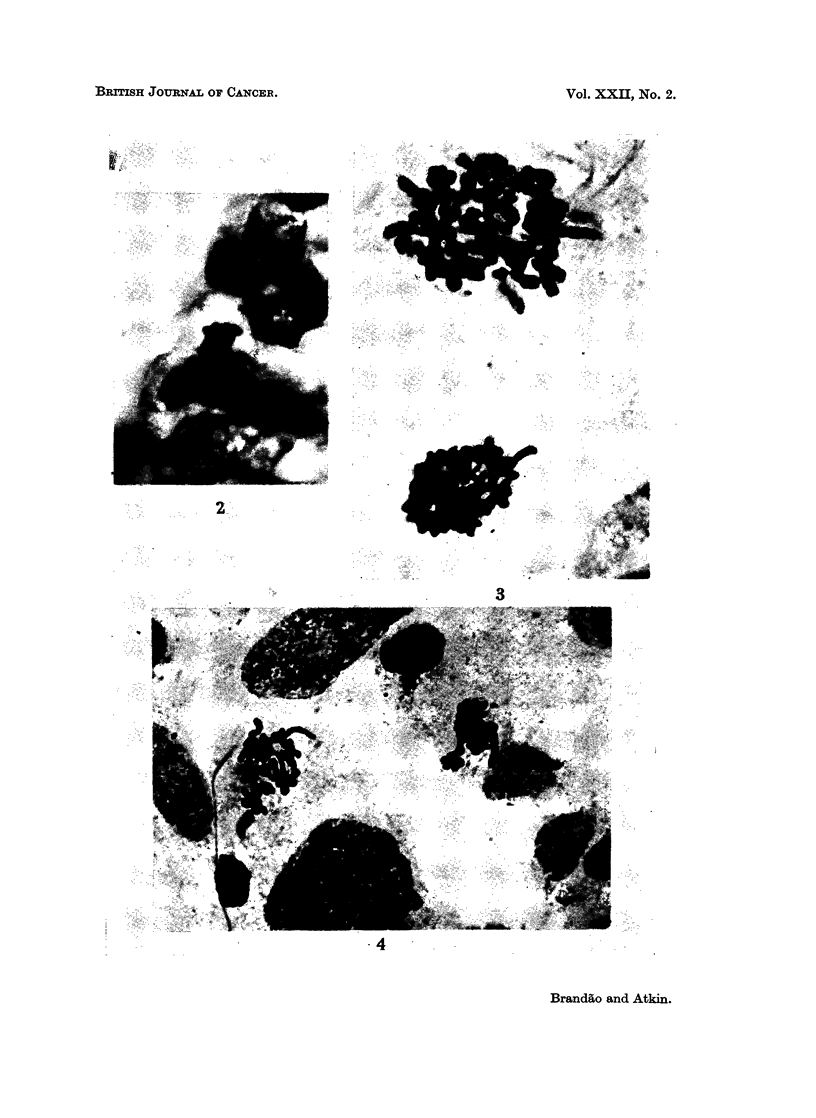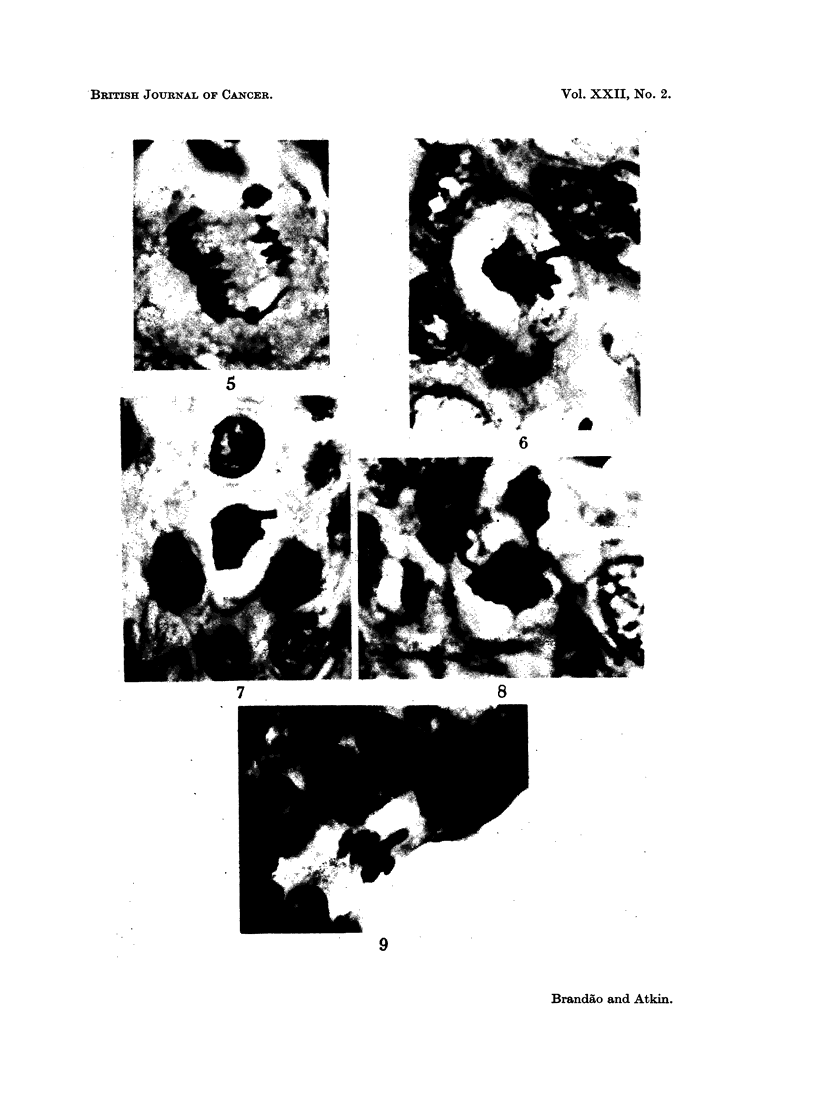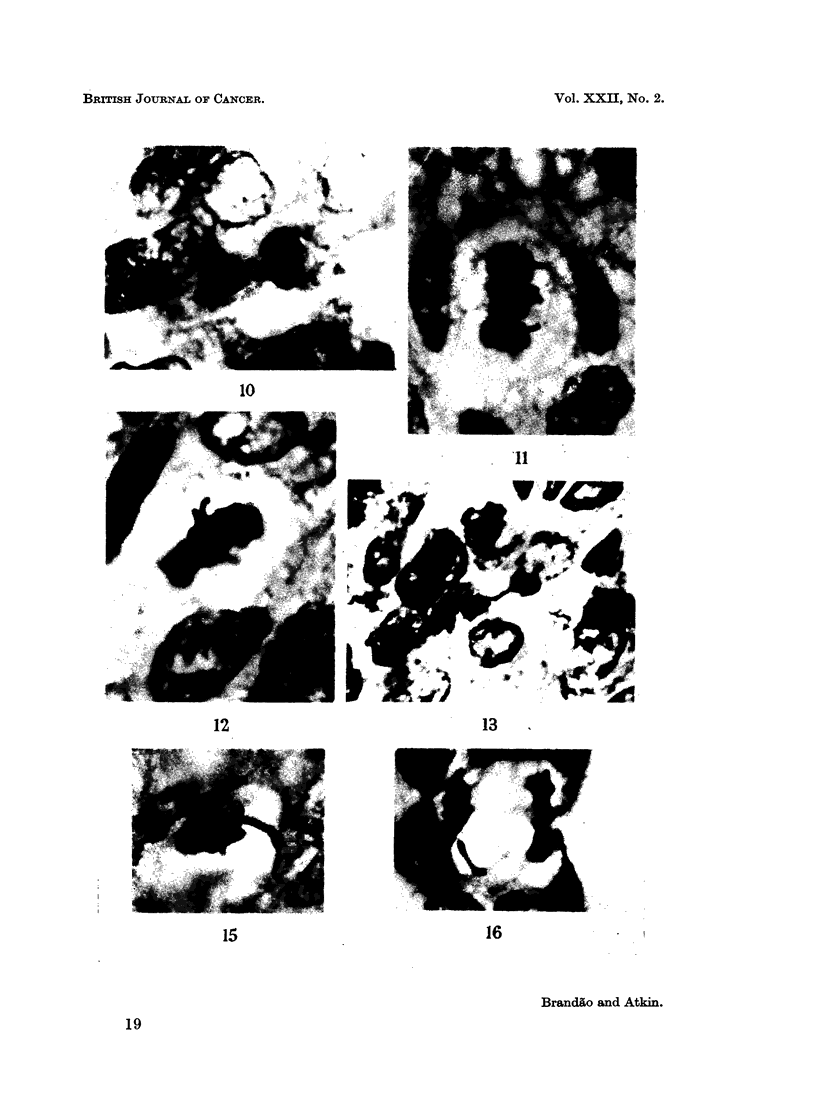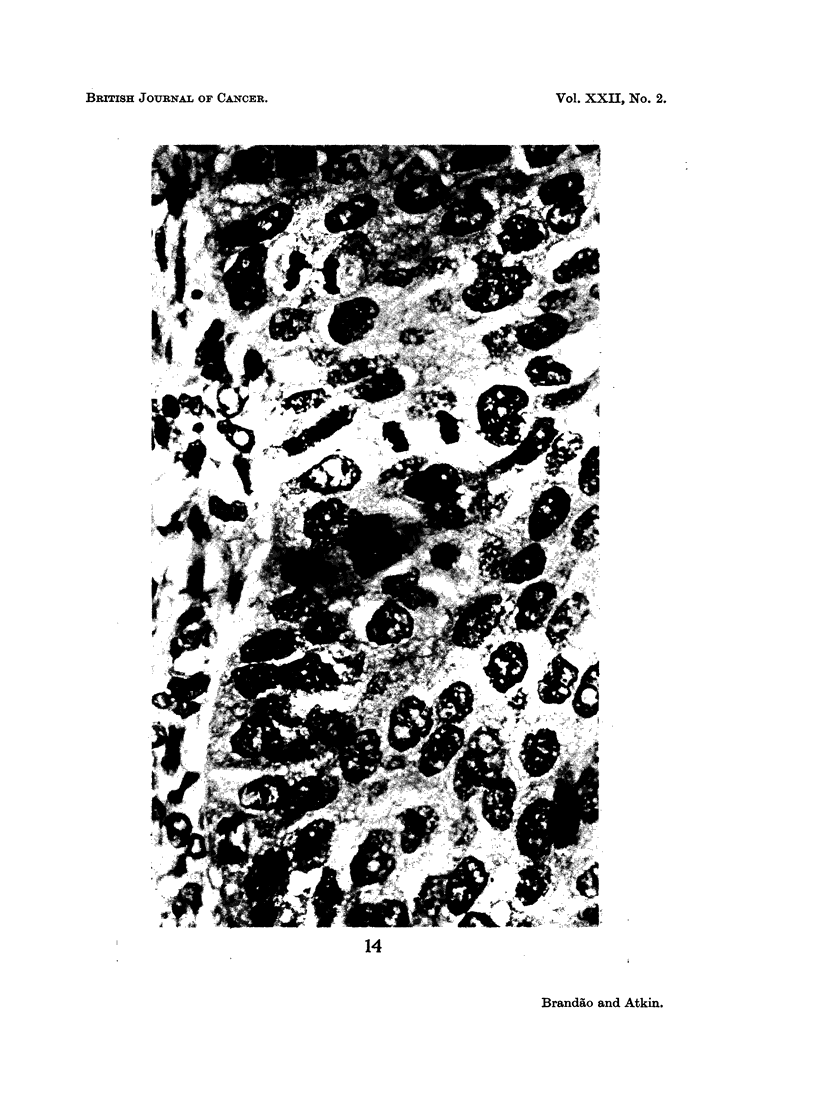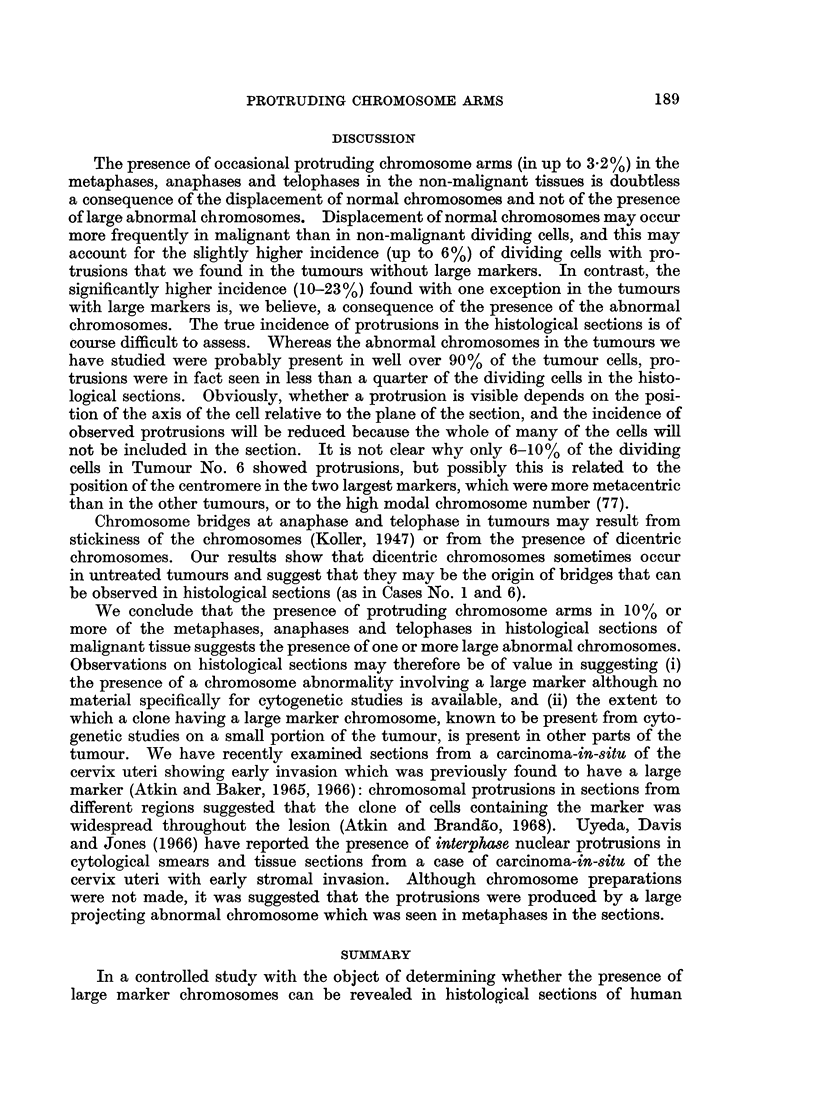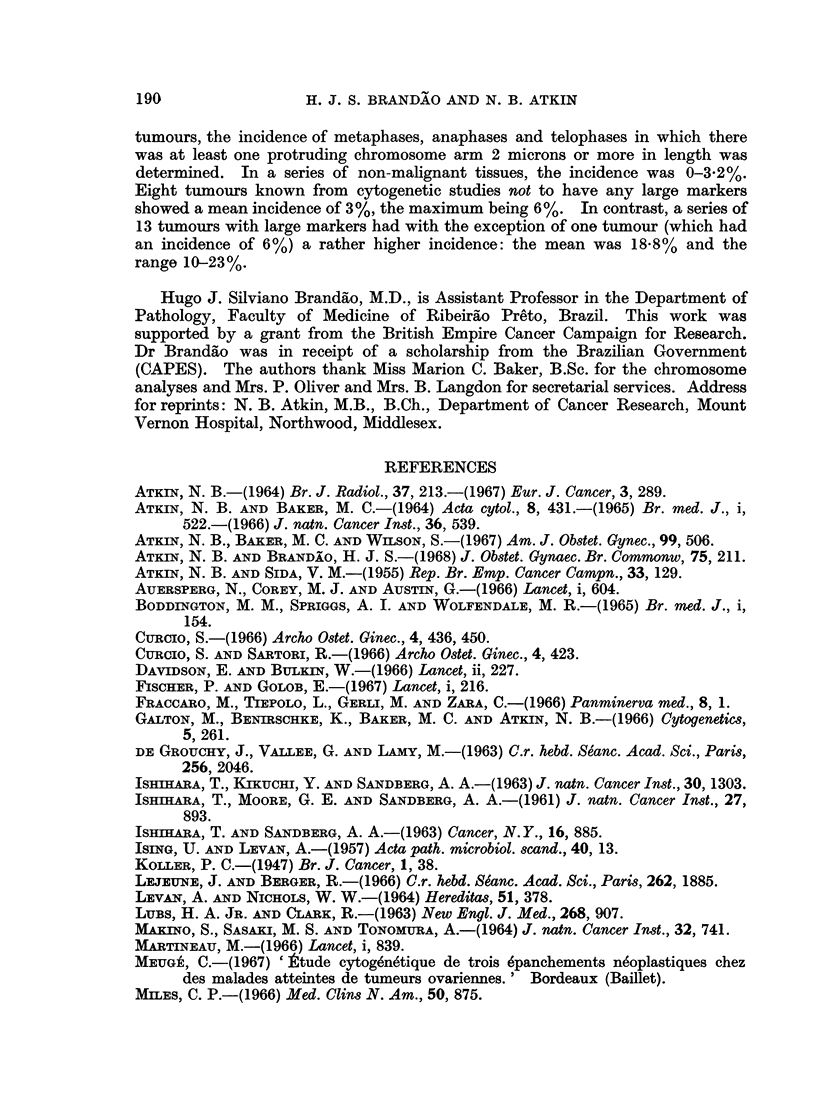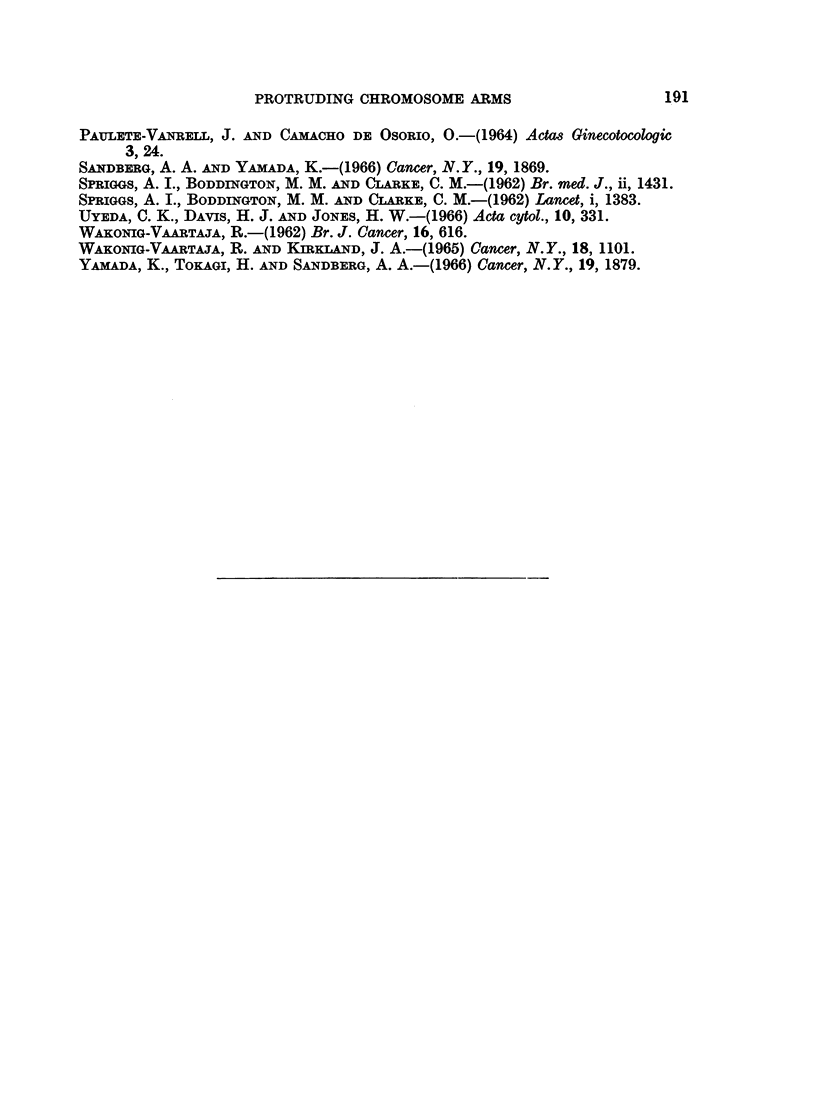# Protruding chromosome arms in histological sections of tumours with large marker chromosomes.

**DOI:** 10.1038/bjc.1968.26

**Published:** 1968-06

**Authors:** H. J. Brandão, N. B. Atkin

## Abstract

**Images:**


### PROTRUDING CHROMOSOME ARMS IN HISTOLOGICAL SECTIONS OF TUMOURS WITH LARGE MARKER CHROMOSOMES

H. J. S. BRANDÃO AND N. B. ATKIN

*From the Department of Cancer Research, Mount Vernon Hospital, Northwood, Middlesex*

Received for publication February 10, 1968

It is now well-recognized that abnormal chromosomes of varying morphology are of frequent occurrence in human cancer cells. In some malignant tumours, one or more large marker chromosomes are found in a high proportion of the metaphases (Atkin, 1964, 1967; Atkin and Baker, 1964, 1966; Atkin, Baker and Wilson, 1967; Atkin and Sida, 1955; Curcio, 1966; Curcio and Sartori, 1966; Davidson and Bulkin, 1966; Fischer and Golob, 1967; Fraccaro, Tiepolo, Gerli and Zara, 1966; Galton, Benirschke, Baker and Atkin, 1966; de Grouchy, Vallee and Lamy, 1963; Ishihara, Kikuchi and Sandberg, 1963; Ishihara, Moore and Sandberg, 1961; Ishihara and Sandberg, 1963; Ising and Levan, 1957; Lejeune and Berger, 1966; Lubs and Clark, 1963; Makino, Sasaki and Tonomura, 1964; Martineau, 1966; Meugé, 1967; Miles, 1966; Paulete-Vanrell and Camacho de Osorio, 1964; Sandberg and Yamada, 1966; Spriggs, Boddington and Clarke, 1962; Wakonig-Vaartaja, 1962; Yamada, Tokagi and Sandberg, 1966). Large marker chromosomes have also been described in dysplasia and carcinoma-*in-situ* of the cervix uteri (Atkin and Baker, 1965; Atkin and Baker, 1966; Auersperg, Corey and Austin, 1966; Boddington, Spriggs and Wolfendale, 1965; Spriggs, Boddington and Clarke, 1962; Wakonig-Vaartaja and Kirkland, 1965). Since the opportunities for obtaining tissue specifically for cytogenetic studies are often limited or non-existent, the possibility that a chromosome abnormality might be revealed by the examination of histological sections seems worth exploring. Although routine histological sections would appear to be unsuitable material for cytogenetic observations, previous findings have suggested that the presence of large marker chromosomes might be revealed in sections: in squash preparations as well as air-dried preparations made for chromosome studies, it was observed that where a large abnormal chromosome is present (having a long arm that is at least as long as the whole of a No. 1 chromosome) its long arm tends to protrude from metaphase, anaphase and telophase chromosome groups, even though the chromosomes as a whole are crowded together (Atkin and Baker, 1964, Fig. 1 and 3). It seemed, therefore, that the long arms of large abnormal chromosomes might also form visible protrusions in sections.

In order to test this hypothesis, we have examined sections from a series of tumours known from cytogenetic studies to have large abnormal chromosomes. These have been compared with a series of tumours which on karyotype analysis were not found to have a large abnormal chromosome, and with a series of non-malignant tissues known or presumed to have a normal karyotype.

### MATERIALS AND METHODS

Twenty-one malignant tumours from adult patients were studied. Details of the tumour sites and histopathological diagnoses are given in Table II. All except Tumours No. 4 and 13 were from female patients. Samples of tissue from each tumour were obtained for chromosome analysis using a direct technique (Atkin and Baker, 1966); at the same time, samples of tissue were sent for routine histological examination. The histological sections, cut at 5 microns and stained with haematoxylin and eosin following fixation in 10% formalin, were kindly made available to us for study by Dr. M. H. Bennett.

In Table II the tumours with one or more large abnormal chromosomes (Cases 1–13) have been listed separately from those without such markers (Cases 14–21). For the purposes of this study, a *large abnormal chromosome* was defined as one whose long arm is at least 1·5 times the mean length of the D-group chromosomes. We selected the D-group chromosomes as the standard of comparison since it seemed that these were usually the most suitable group of normal chromosomes in the tumour-cell karyotypes, being easily recognizable and of reasonable length. In normal cells the ratio between the longest long arms (those of the A2 chromosomes) and the mean length of the D-group chromosomes, from measurements given in the report of the Denver conference (Levan and Nichols, 1964), is of the order of 1·47. We measured apparently normal A2 and D-group chromosomes in 12 metaphases from 4 of the group of tumours in the present series which presented no structurally abnormal chromosomes, and found a mean ratio of 1·27 (standard deviation $\pm 0{\cdot}08$).

All measurements were made on photographic enlargements printed at $\times 4000$. Metaphases suitable for measurement were not obtained from Cases 4 and 5, but observations on orcein squash preparations clearly showed the presence of large abnormal chromosomes (Fig. 3 and 4). Measurements were made on 1 to 10 metaphases from each tumour. Single representative metaphases were measured where there was little doubt from the examination of a number of metaphases that an abnormal chromosome was present whose long arm clearly exceeded that of the No. 2 chromosome. As indicated in Table II, some tumours had more than one large marker. Measurements were made on the *long arm* of the abnormal chromosome because it seemed likely that its length, rather than the total length of the chromosome, would determine whether a protrusion was seen in dividing cells in the histological sections.

The histological sections were scanned using a $100\times$ oil-immersion objective and where chromosome arms protruded from the dividing cells, the length of the protrusions were measured with the aid of a calibrated eyepiece micrometer.

In the control study, 758 metaphases, anaphases and telophases from 9 non-malignant tissues (endometrium, lymph node and appendix) were assessed (Table I). On karyotype analysis, no chromosome abnormalities were found in direct preparations of the endometria or in a short-term culture of the lymph node; chromosome analysis was not performed on the appendix material.

Preliminary examination of the sections of non-malignant tissues showed that although small protruding chromosome arms were quite frequently seen, protrusions of 2 microns or more in length were seldom encountered. Protrusions were therefore only recorded if they were at least 2 microns in length.

Altogether, 1942 dividing cells from the sections of tumour tissue were assessed. The number of cells assessed for each tumour varied from 38 to 100. Mitoses

TABLE I.—*Non-neoplastic Tissues: Incidence of Metaphases, Anaphases and Telophases with Protruding Chromosome Arms in Histological Sections*

| Case No. | Age | Type of tissue | Percentage of dividing cells with protruding chromosome arms (in brackets: number of cells assessed) | |
|---|---|---|---|---|
| 1 | 37 | Epithelium and stroma of normal endometrium | 2·0% | (50) |
| 2 | 30 | Epithelium and stroma of normal endometrium | 3·0% | (100) |
| 3 | 44 | Epithelium and stroma of normal endometrium | 2·0% | (100) |
| 4 | 45 | Epithelium and stroma of normal endometrium | 0% | (100) |
| 5 | 44 | Epithelium and stroma of benign hyperplastic endometrium | 1·1% | (92) |
| 6 | 71 | Epithelium and stroma of benign hyperplastic endometrium | 3·2% | (125) |
| 7 | 22 | Germinal centre of lymph node showing benign reactive hyperplasia (female) | 1·0% | (100) |
| 8 | 52 | Epithelium of vermiform appendix (female) | 0% | (40) |
| 9 | 27 | Epithelium of vermiform appendix (male) | 0% | (50) |

having chromosome groups with very irregular outlines, or with scattered chromosomes lying apart from the main group, were excluded. To avoid bias, the histological sections were assessed at random by one of the authors (H.J.S.B.), without prior knowledge of the cytogenetic findings.

Illustrations or descriptions of karyotypes from some of the tumours have appeared elsewhere:

| Case No. | No. given to the case in the previous publication | Reference |
|---|---|---|
| 2 | — | Atkin, 1967 |
| 6 | 4 | Atkin, Baker, and Wilson, 1967 |
| 7 | 3 | ,, |
| 15 | 1 | ,, |
| 21 | 2 | ,, |
| 3 | 7 | Atkin and Baker, 1966 |
| 8 | 3 | ,, |
| 11 | 2 | ,, |
| 12 | 4 | ,, |
| 16 | 16 | ,, |
| 17 | 10 | ,, |
| 20 | 14 | ,, |

### RESULTS

The incidence of metaphases, anaphases and telophases in which there were chromosomal protrusions 2 microns or more in length in the 9 non-neoplastic tissues is shown in Table I and in the 21 tumours in Table II. In the non-neoplastic tissues the highest incidence was 3·2%, the mean being 1·3%. The tumours *without* long markers (Table II, Cases 14–21) showed an incidence which on the whole slightly exceeded that found in the non-malignant tissues: the highest incidence was 6·0% and the mean was 3·0%.

On the other hand, the tumours which were known from cytogenetic studies to have at least one large abnormal chromosome (Table II, Cases 1–13) showed with one exception (Case No. 6) a significantly higher incidence of protrusions. Excluding Case No. 6, the mean incidence was 18·8% and the range was 10–23%. Although Case No. 6, which had 2 large submetacentric markers, showed a low incidence of cells having protrusions, some metaphases clearly had two protru-

TABLE II.—*Malignant Tumours: Cytogenetic Observations and Incidence of Metaphases, Anaphases and Telophases with Protruding Chromosome Arms 2 Microns or More in Length in Histological Sections*

| | | | Karyotype analysis | | | | Observations on histological sections | |
|---|---|---|---|---|---|---|---|---|
| Case No. | Age | Histopathological diagnosis | Length of long arm of the largest abnormal chromosome relative to the mean length of the D-group chromosomes (in brackets: number of cells measured) | | Number of large abnormal chromosomes (in brackets: number in cells with high chromosome numbers, where two modes present) | Modal chromosome number(s) | Percentage of dividing cells with protruding chromosome arms (in brackets: number of cells assessed) | |
| 1 | 62 | Poorly differentiated spheroidal cell carcinoma of the breast | 2·6 | (4) | 3 | 41 | 14% | (100) |
| 2 | 77 | Poorly differentiated adenoacanthoma of the cervix uteri | 2·1 | (1) | 3 (6) | 40 and 81 | 19% | (100) |
| 3 | 49 | Poorly differentiated squamous cell carcinoma of the cervix uteri | 2·2 | (3) | 1 | 41 | 23% | (100) |
| 4 | 56 | Poorly differentiated squamous cell carcinoma of the bladder | — | — | — | — | 23% | (70) |
| 5 | 64 | Poorly differentiated transitional cell carcinoma of the bladder | — | — | — | — | 10% | (58) |
| 6 | 54 | Poorly differentiated squamous cell carcinoma of the cervix uteri | 1·8 | (3) | 3 | 77 | 6% | (100) |
| 7 | 59 | Poorly differentiated squamous cell carcinoma of the cervix uteri | 2·6 | (1) | 1 | 44 | 17% | (100) |
| 8 | 41 | Poorly differentiated squamous cell carcinoma of the cervix uteri | 1·7 | (10) | 1 | 60 | 13% | (100) |
| 9 | 63 | Moderately well differentiated papillary adenocarcinoma of ovarian origin (secondary in omentum) | 2·4 | (3) | 3 | 59 | 20% | (100) |
| 10 | 62 | Poorly differentiated adenocarcinoma of the endometrium | 1·6 | (5) | 1 (2) | 41 and 82 | 23% | (38) |
| 11 | 55 | Moderately well differentiated papillary adenocarcinoma suggestive of ovarian origin (lymph node) | 2·4 | (1) | 2 | 54 | 22% | (100) |
| 12 | 88 | Reticulum cell sarcoma (lymph node) | 1·8 | (1) | 2 | 49 | 15% | (100) |
| 13 | 57 | Well differentiated columnar cell adenocarcinoma of the colon | 2·0 | (3) | 1 | 46 | 21% | (100) |
| 14 | 52 | Moderately well differentiated columnar cell adenocarcinoma of the endometrium | — | — | — | 47 | 4% | (100) |
| 15 | 58 | Poorly differentiated squamous cell carcinoma of the cervix uteri | — | — | — | 48 | 3% | (100) |
| 16 | 50 | Moderately well differentiated columnar cell carcinoma of the endometrium | — | — | — | 53 | 3% | (100) |
| 17 | 57 | Undifferentiated adenocarcinoma of the endometrium | — | — | — | 46 | 2% | (100) |
| 18 | 81 | Moderately well differentiated mucus-secreting columnar cell adenocarcinoma of the colon | — | — | — | 46 | 0% | (80) |
| 19 | 73 | Well differentiated papillary columnar cell adenocarcinoma of the endometrium | — | — | — | 46 | 4% | (100) |
| 20 | 75 | Moderately well differentiated colloid carcinoma of the colon | — | — | — | 51 | 2% | (100) |
| 21 | 84 | Moderately well differentiated squamous cell carcinoma of the cervix uteri | — | — | — | 47 | 6% | (100) |

sions, and some anaphases and telophases four protrusions (Fig. 10, 11 and 12). Chromosome bridges were also seen (Fig. 14); these may have been produced by a dicentric chromosome which was found in about 25% of the metaphases in the cytogenetic studies (Atkin, Baker, and Wilson, 1967). In view of the anomalous findings on Case No. 6, further counts were made on two different regions; these showed incidences of 8% and 10% of cells with protrusions respectively. Examples of protrusions in dividing cells from some of the other tumours are shown in Fig. 5–9. The karyotype of a metaphase in a cytogenetic preparation from Case No. 1 is shown in Fig. 1.

Occasionally, the whole of a large abnormal chromosome could be seen, where it had become separated from the other chromosomes (Fig. 15 and 16); in the metaphase from Case No. 3 (Fig. 15) this chromosome was clearly seen to be a subacrocentric chromosome similar to that found in the cytogenetic preparations.

In addition to Case No. 6, a dicentric was found in some of the metaphases from Case No. 1, and chromosome bridges were seen at anaphase and telophase in the histological sections (Fig. 13).

Neither the modal chromosome numbers of the tumours, which varied over a fairly wide range (Table II), nor the presence in some tumours of more than one large abnormal chromosome appeared to show any correlation with the incidence of cells with protrusions in the histological sections. The incidence of anaphases with protrusions (25·2% of all anaphases assessed from Cases 1–13) was higher than the incidence of metaphases with protrusions (10·2%).

---

### EXPLANATION OF PLATES

FIG. 1.—Karyotype of metaphase from Tumour No. 1 (carcinoma of breast), ×3000. A dicentric chromosome, which was seen in some of the metaphases from this tumour, is not present in this cell. 41 chromosomes; M = large marker.

FIG. 2.—Control Case No. 2. Protruding chromosome arm in a metaphase from normal endometrium. Histological section, H. and E. ×1950.

FIG. 3 and 4.—Orcein squash preparations of tumour tissue showing metaphases with a protruding abnormal chromosome. Fig. 3: Case No. 4 (carcinoma of bladder), ×2000. Fig. 4: Case No. 5 (carcinoma of bladder), ×1050.

FIG. 5.—Case No. 4 (carcinoma of bladder). Anaphase showing delayed separation of the chromatids of a large abnormal chromosome. Histological section, H. and E. ×1900.

FIG. 6 and 7.—Case No. 7 (carcinoma of cervix uteri). Metaphases showing protruding arm of a large abnormal chromosome. Histological section, H. and E. ×1900.

FIG. 8.—Case No. 3 (carcinoma of cervix uteri). Metaphase showing protruding arm of a large abnormal chromosome. Histological section, H. and E. ×1900.

FIG. 9.—Case No. 9 (carcinoma of ovary). Metaphase showing protruding arm of a large abnormal chromosome. Histological section, H. and E. ×1900.

FIG. 10.—Case No. 6 (carcinoma of cervix uteri). Late anaphase showing two chromosome arms protruding from each daughter chromosome group. Histological section, H. and E. ×1950.

FIGS. 11 and 12.—Case No. 6 (carcinoma of cervix uteri). Two metaphases, each showing two protruding chromosome arms. Histological section, H. and E. ×1950.

FIG. 13.—Case No. 1 (carcinoma of breast). Telophase showing chromosome bridge. Histological section, H. and E. ×1250.

FIG. 14.—Case No. 6 (carcinoma of cervix uteri). Late anaphase (lower left) showing bridge; telophase (top left) showing two chromosomal protrusions. Histological section, H. and E. ×770.

FIG 15.—Case No. 3 (carcinoma of cervix uteri). The whole of a large abnormal chromosome is seen lying to the right of the main metaphase chromosome group. Histological section, H. and E. ×1950.

FIG. 16.—Case No. 9 (carcinoma of ovary). Late anaphase showing the whole of a lagging large abnormal chromosome. Histological section, H. and E. ×1950.

Brandão and Atkin.

2

3

4

5

6

7

8

9

Brandão and Atkin.

10

11

12

13

15

16

14

Brandão and Atkin.

### DISCUSSION

The presence of occasional protruding chromosome arms (in up to 3·2%) in the metaphases, anaphases and telophases in the non-malignant tissues is doubtless a consequence of the displacement of normal chromosomes and not of the presence of large abnormal chromosomes. Displacement of normal chromosomes may occur more frequently in malignant than in non-malignant dividing cells, and this may account for the slightly higher incidence (up to 6%) of dividing cells with protrusions that we found in the tumours without large markers. In contrast, the significantly higher incidence (10–23%) found with one exception in the tumours with large markers is, we believe, a consequence of the presence of the abnormal chromosomes. The true incidence of protrusions in the histological sections is of course difficult to assess. Whereas the abnormal chromosomes in the tumours we have studied were probably present in well over 90% of the tumour cells, protrusions were in fact seen in less than a quarter of the dividing cells in the histological sections. Obviously, whether a protrusion is visible depends on the position of the axis of the cell relative to the plane of the section, and the incidence of observed protrusions will be reduced because the whole of many of the cells will not be included in the section. It is not clear why only 6–10% of the dividing cells in Tumour No. 6 showed protrusions, but possibly this is related to the position of the centromere in the two largest markers, which were more metacentric than in the other tumours, or to the high modal chromosome number (77).

Chromosome bridges at anaphase and telophase in tumours may result from stickiness of the chromosomes (Koller, 1947) or from the presence of dicentric chromosomes. Our results show that dicentric chromosomes sometimes occur in untreated tumours and suggest that they may be the origin of bridges that can be observed in histological sections (as in Cases No. 1 and 6).

We conclude that the presence of protruding chromosome arms in 10% or more of the metaphases, anaphases and telophases in histological sections of malignant tissue suggests the presence of one or more large abnormal chromosomes. Observations on histological sections may therefore be of value in suggesting (i) the presence of a chromosome abnormality involving a large marker although no material specifically for cytogenetic studies is available, and (ii) the extent to which a clone having a large marker chromosome, known to be present from cytogenetic studies on a small portion of the tumour, is present in other parts of the tumour. We have recently examined sections from a carcinoma-*in-situ* of the cervix uteri showing early invasion which was previously found to have a large marker (Atkin and Baker, 1965, 1966): chromosomal protrusions in sections from different regions suggested that the clone of cells containing the marker was widespread throughout the lesion (Atkin and Brandão, 1968). Uyeda, Davis and Jones (1966) have reported the presence of *interphase* nuclear protrusions in cytological smears and tissue sections from a case of carcinoma-*in-situ* of the cervix uteri with early stromal invasion. Although chromosome preparations were not made, it was suggested that the protrusions were produced by a large projecting abnormal chromosome which was seen in metaphases in the sections.

### SUMMARY

In a controlled study with the object of determining whether the presence of large marker chromosomes can be revealed in histological sections of human tumours, the incidence of metaphases, anaphases and telophases in which there was at least one protruding chromosome arm 2 microns or more in length was determined. In a series of non-malignant tissues, the incidence was 0–3·2%. Eight tumours known from cytogenetic studies *not* to have any large markers showed a mean incidence of 3%, the maximum being 6%. In contrast, a series of 13 tumours with large markers had with the exception of one tumour (which had an incidence of 6%) a rather higher incidence: the mean was 18·8% and the range 10–23%.

Hugo J. Silviano Brandão, M.D., is Assistant Professor in the Department of Pathology, Faculty of Medicine of Ribeirão Prêto, Brazil. This work was supported by a grant from the British Empire Cancer Campaign for Research. Dr Brandão was in receipt of a scholarship from the Brazilian Government (CAPES). The authors thank Miss Marion C. Baker, B.Sc. for the chromosome analyses and Mrs. P. Oliver and Mrs. B. Langdon for secretarial services. Address for reprints: N. B. Atkin, M.B., B.Ch., Department of Cancer Research, Mount Vernon Hospital, Northwood, Middlesex.

### REFERENCES

ATKIN, N. B.—(1964) *Br. J. Radiol.*, **37**, 213.—(1967) *Eur. J. Cancer*, **3**, 289.

ATKIN, N. B. AND BAKER, M. C.—(1964) *Acta cytol.*, **8**, 431.—(1965) *Br. med. J.*, i, 522.—(1966) *J. natn. Cancer Inst.*, **36**, 539.

ATKIN, N. B., BAKER, M. C. AND WILSON, S.—(1967) *Am. J. Obstet. Gynec.*, **99**, 506.

ATKIN, N. B. AND BRANDÃO, H. J. S.—(1968) *J. Obstet. Gynaec. Br. Commonw*, **75**, 211.

ATKIN, N. B. AND SIDA, V. M.—(1955) *Rep. Br. Emp. Cancer Campn.*, **33**, 129.

AUERSPERG, N., COREY, M. J. AND AUSTIN, G.—(1966) *Lancet*, i, 604.

BODDINGTON, M. M., SPRIGGS, A. I. AND WOLFENDALE, M. R.—(1965) *Br. med. J.*, i, 154.

CURCIO, S.—(1966) *Archo Ostet. Ginec.*, **4**, 436, 450.

CURCIO, S. AND SARTORI, R.—(1966) *Archo Ostet. Ginec.*, **4**, 423.

DAVIDSON, E. AND BULKIN, W.—(1966) *Lancet*, ii, 227.

FISCHER, P. AND GOLOB, E.—(1967) *Lancet*, i, 216.

FRACCARO, M., TIEPOLO, L., GERLI, M. AND ZARA, C.—(1966) *Panminerva med.*, **8**, 1.

GALTON, M., BENIRSCHKE, K., BAKER, M. C. AND ATKIN, N. B.—(1966) *Cytogenetics*, **5**, 261.

DE GROUCHY, J., VALLEE, G. AND LAMY, M.—(1963) *C.r. hebd. Séanc. Acad. Sci., Paris*, **256**, 2046.

ISHIHARA, T., KIKUCHI, Y. AND SANDBERG, A. A.—(1963) *J. natn. Cancer Inst.*, **30**, 1303.

ISHIHARA, T., MOORE, G. E. AND SANDBERG, A. A.—(1961) *J. natn. Cancer Inst.*, **27**, 893.

ISHIHARA, T. AND SANDBERG, A. A.—(1963) *Cancer, N.Y.*, **16**, 885.

ISING, U. AND LEVAN, A.—(1957) *Acta path. microbiol. scand.*, **40**, 13.

KOLLER, P. C.—(1947) *Br. J. Cancer*, **1**, 38.

LEJEUNE, J. AND BERGER, R.—(1966) *C.r. hebd. Séanc. Acad. Sci., Paris*, **262**, 1885.

LEVAN, A. AND NICHOLS, W. W.—(1964) *Hereditas*, **51**, 378.

LUBS, H. A. JR. AND CLARK, R.—(1963) *New Engl. J. Med.*, **268**, 907.

MAKINO, S., SASAKI, M. S. AND TONOMURA, A.—(1964) *J. natn. Cancer Inst.*, **32**, 741.

MARTINEAU, M.—(1966) *Lancet*, i, 839.

MEUGÉ, C.—(1967) 'Étude cytogénétique de trois épanchements néoplastiques chez des malades atteintes de tumeurs ovariennes.' Bordeaux (Baillet).

MILES, C. P.—(1966) *Med. Clins N. Am.*, **50**, 875.

PAULETE-VANRELL, J. AND CAMACHO DE OSORIO, O.—(1964) *Actas Ginecotocologic* **3**, 24.
SANDBERG, A. A. AND YAMADA, K.—(1966) *Cancer, N.Y.*, **19**, 1869.
SPRIGGS, A. I., BODDINGTON, M. M. AND CLARKE, C. M.—(1962) *Br. med. J.*, ii, 1431.
SPRIGGS, A. I., BODDINGTON, M. M. AND CLARKE, C. M.—(1962) *Lancet*, i, 1383.
UYEDA, C. K., DAVIS, H. J. AND JONES, H. W.—(1966) *Acta cytol.*, **10**, 331.
WAKONIG-VAARTAJA, R.—(1962) *Br. J. Cancer*, **16**, 616.
WAKONIG-VAARTAJA, R. AND KIRKLAND, J. A.—(1965) *Cancer, N.Y.*, **18**, 1101.
YAMADA, K., TOKAGI, H. AND SANDBERG, A. A.—(1966) *Cancer, N.Y.*, **19**, 1879.